# Ensembles of knowledge graph embedding models improve predictions for drug discovery

**DOI:** 10.1093/bib/bbac481

**Published:** 2022-11-16

**Authors:** Daniel Rivas-Barragan, Daniel Domingo-Fernández, Yojana Gadiya, David Healey

**Affiliations:** Enveda Biosciences, Boulder, CO, USA; Enveda Biosciences, Boulder, CO, USA; Enveda Biosciences, Boulder, CO, USA; Enveda Biosciences, Boulder, CO, USA

**Keywords:** drug discovery, link prediction, machine learning, knowledge graph embedding models, knowledge graphs, ensemble learning

## Abstract

Recent advances in Knowledge Graphs (KGs) and Knowledge Graph Embedding Models (KGEMs) have led to their adoption in a broad range of fields and applications. The current publishing system in machine learning requires newly introduced KGEMs to achieve state-of-the-art performance, surpassing at least one benchmark in order to be published. Despite this, dozens of novel architectures are published every year, making it challenging for users, even within the field, to deduce the most suitable configuration for a given application. A typical biomedical application of KGEMs is drug–disease prediction in the context of drug discovery, in which a KGEM is trained to predict triples linking drugs and diseases. These predictions can be later tested in clinical trials following extensive experimental validation. However, given the infeasibility of evaluating each of these predictions and that only a minimal number of candidates can be experimentally tested, models that yield higher precision on the top prioritized triples are preferred. In this paper, we apply the concept of ensemble learning on KGEMs for drug discovery to assess whether combining the predictions of several models can lead to an overall improvement in predictive performance. First, we trained and benchmarked 10 KGEMs to predict drug–disease triples on two independent biomedical KGs designed for drug discovery. Following, we applied different ensemble methods that aggregate the predictions of these models by leveraging the distribution or the position of the predicted triple scores. We then demonstrate how the ensemble models can achieve better results than the original KGEMs by benchmarking the precision (i.e., number of true positives prioritized) of their top predictions. Lastly, we released the source code presented in this work at https://github.com/enveda/kgem-ensembles-in-drug-discovery.

## Introduction

Applications of knowledge graphs (KGs) are steadily increasing in the biomedical domain, including the prediction of side effects of a given drug in the early stages of drug development [1], drug repositioning [2] and target prioritization [3]. Several approaches have previously been proposed for biomedical applications using KGs. These include meta-path-based approaches which leverage a relevant type of path in a network (e.g., drug–protein–disease for drug discovery) [4], node similarity-based approaches which exploit network properties (e.g., shared neighbors or other connectivity features) [5], path reasoning-based approaches which take edge types within a path into account [6] and machine learning-based approaches in which a model learns node and relation embeddings that are subsequently used for predicting relevant connections, such as gene–disease associations and drug-targets [7].

Recently, parallel to the adoption of KGs for applications through wide-reaching domains, a substantial number of machine learning based approaches have also been developed, such as Knowledge Graph Embedding Models (KGEMs) [8]. These models are trained to learn a low-dimensional representation of the entities and relations in a KG so that the KG can be exploited for link prediction, among other applications. The goal of link prediction is to predict new triples or infer missing ones between non-connected nodes within a network. Although for most non-biomedical KGs one may want to optimize the model to learn all relations present in the KG in equal proportions (e.g., predicting heterogeneous triples in social media networks), biomedical KGs typically entail that the model is focused on a specific relation type which corresponds to a biomedical application (e.g., drug–disease for drug discovery, protein–disease for target prioritization and drug-side effect for side effect prediction) [9]. This distinct condition typically required by applications of biomedical KGs can adversely affect the performance of KGEMs as the majority of these models have been benchmarked for non-biomedical KGs which require the simultaneous optimization of the model to predict all or multiple relation types [10].

To remedy this problem, one can restrict the validation and test set to a particular relation type, thereby reinforcing the training of the KGEM towards the relation type of interest. However, such a training procedure could potentially be detrimental or even infeasible depending on the characteristics of the KG, for example, in drug discovery applications where the proportion of drug–disease relations is minimal compared with other relations. Furthermore, KGEMs predictions can vary depending on several factors such as data splitting (e.g., negative triple generation and train-validation-test splits) and the hyperparameters of the model [11]. Finally, given that dozens of KGEMs have been published so far but have only rarely been benchmarked on biomedical KGs, KGEM selection plays a significant role in the final predictions. Especially, due to their topological differences with the classical benchmark KGs such as FB15k and WikiData. As a consequence, two KGEMs trained on the same biomedical KG can result in a completely disjoint set of predictions [12], even if each of these predictions may be correct.

One of the major biomedical applications of link prediction using KGEMs is drug–disease prioritization for drug discovery [5]. By predicting triples on a set of chemicals for a given disease, KGEMs can quickly nominate a set of compounds that could be experimentally tested. This prioritization is extremely valuable given the size of the chemical space, and allows focusing on a limited number of chemicals, thereby increasing the chances of success in clinical trials as well as reducing the time and cost associated with drug discovery. However, the aforementioned challenges related with KGEMs can introduce variability in the prediction scores among the top-predicted triples. Furthermore, given the embedding spaces generated by each model and thus, distinct score distributions, the predictions from one model cannot directly be compared with the predictions from another without any normalization; thus, relying on a single KGEM. In our work, we start by comparing the performance of a variety of KGEMs on two KGs designed for drug discovery. In line with previous work that investigated biomedical KGs, our results show great variability across models. Finally, we also investigate whether the performance of these models can be improved by leveraging an ensemble of models.

## Related work

Recent benchmarks have investigated the performance of KGEMs in biomedical KGs. Chang *et al.* [13] conducted an evaluation of five models on SNOMED-CT, an immense KG with over 2 000 000 triples (170 edge types) and ~300 000 nodes. In addition, Bonner *et al*. [14] conducted a benchmark on two large biomedical KGs using five models. Apart from a general link prediction task for any type of edge as the previous work by Chang *et al.*, the authors trained the models to specifically predict gene–disease triples. However, the characteristic of these benchmarked KGs impeded benchmarking the predictions of KGEMs on drug–disease triples.

Ensemble learning is a widely used approach that combines predictions from multiple models with the goal of achieving a better performance than any of the individual models. This concept was first explored in the context of KGEMs by Krompaß and Tresp [15], when only a handful of KGEMs had been published. In their work, the authors proposed an ensemble model where they aggregate scores of three models (i.e., RESCAL [16], TransE [17] and ERMLP [18]). To harmonize the disparate score distributions generated by each model, they transformed them using a Platt-Scaler model [19] learned from a subset of the triples to subsequently aggregate them to generate the ensemble scores. They then demonstrated that the predictions of the ensemble were more accurate than ones from the individual models in three benchmark KGs. More recently, Choi *et al*. [20] presented a similar concept in which instead of using a subset of the triples, the entire distribution of triple scores is normalized to be subsequently leveraged by a Product of Experts (PoE) to yield ensemble predictions [21]. Here, the authors employ four translational distance models to also demonstrate that the ensemble outperforms the individual models in two KGs. Finally, Xu *et al.* [22] also showed on the same KGs, how parallely training the same model with a low dimension and combining their scores achieves a better performance than training the corresponding model with a higher dimension.

Taken together, aforementioned studies have demonstrated the potential of ensemble-based approaches using KGEMs. Although prior work has focused on five translational distance KGEMs and benchmark KGs such as FB15k and WN18, it is still open to what extends ensemble models can improve the predictions in biomedical KGs designed for drug discovery given their particular characteristics (e.g., link prediction is conducted on a specific relation, this relation in a minority of the triples, smaller number of relation types, nodes, triples, etc.). Given the large number of novel KGEMs published in the last years, and the recently released libraries for training, it is now possible to explore the performance of ensemble models on a variety of model configurations.

## Methods

### KGEMs

We employed 10 different KGEMs: RESCAL [16], TransE [17], DistMult [23], ERMLP [18], TransH [24], ComplEx [25], HolE [26], ConvE [27], RotatE [28] and MuRE [29]. These models have been selected based on: (i) their variability in terms of modeling paradigms [11], (ii) their performance on benchmarks [11] and (iii) their prior use for applications in drug discovery [10, 30, 31]. Supplementary Table 1 summarizes the key properties of the models.

### KGs

We benchmarked two KGs: BioKG [32] and OpenBioLink [33]. Since both KGs are designed for a variety of biomedical applications (e.g., drug repurposing and side effect predictions), they contain different node (e.g., proteins, phenotypes and anatomical regions) and relation types (e.g., inhibition, activation and binding in OpenBioLink, and protein–protein interactions and drug–drug interactions in BioKG) that were normalized in the steps outlined below.

In the first normalization step, the two KGs were reduced to three types of nodes: drugs, proteins and diseases. Modeling both KGs this way allows for better comparison, as we used an equivalent schema to represent the mechanism of action (MoA) of a drug (i.e., a drug binds a target and leads to a cascade of events that revert the pathophysiology of an indication). Furthermore, some of the node types removed (i.e., protein functions and side effects for BioKG, and phenotypes, pathways, anatomy, and biological function for OpenBioLink) can also be used as node properties (e.g., protein function) and are present in the KG for other biomedical applications (e.g., side effect prediction). For instance, in a recent drug repurposing study using KGEMs, the authors showed how filtering out entities that do not appear in metapaths connecting chemicals and diseases significantly improved the performance of the models [10]. This study showed that after this filtering, although the relative frequency of chemicals and diseases nodes increased, the frequency of other node types, such as pathway or cellular component nodes (the ones we removed) was significantly reduced. Lastly, we would like to note the importance of reducing the original size of the KGs to achieve a reasonable computational time to train the KGEMs. The training of some KGEMs on NVIDIA V100 GPUs with 32 GB of memory required several days, and the addition, for example, of the 1 million drug–drug interactions present in BioKG alone would exponentially increase the computational time of training the model.

In the second normalization step, the original relations types of the KG were maintained with the exception of protein–protein interactions in OpenBioLink as only causal relations were considered (i.e., activates and inhibits). Supplementary Figure 1 shows the statistics for each KG, at both the node and relation levels.

### Train, validation and test datasets

For training and evaluating the KGEMs, we split the two KGs into train, validation and test sets. Since we ultimately aim at predicting drug–disease triples, the validation and test datasets exclusively contain this type of edge, whereas the train dataset contains all edge types (i.e. drug–disease, drug–protein, protein–protein and protein–disease). Furthermore, similar to Ratajczak *et al*. [10], we distributed the drug–disease triples along the three splits with ~80% of the drug–disease triples in the train, ~10% in the validation and ~10% in the test split. Accordingly, the overall split ratio for all triples is ~96, 2 and 2% for the train, validation and test datasets, respectively. Table 1 provides the comparative summary of the drug and disease triples between the three splits for each KG. Finally, it is important to note that all drug–disease triples are directed and thus, inverse triples were not considered as previous benchmarks show that their inclusion degrades performance [12].

**Table 1:** Distribution of drug–disease triples across the train, validation and test splits

**KG**	**Train (%)**	**Validation (%)**	**Test (%)**
**BioKG**	41 648 (80%)	5206 (10%)	5206 (10%)
**OpenBioLink**	4108 (80%)	516 (10%)	515 (10%)

### Implementation

KGEMs have been trained using the PyKEEN framework (v1.8.0) [34]. All experiments were performed on machines with Intel(R) Xeon(R) Gold 5218 CPUs and 8 NVIDIA Tesla V100 32 GB GPUs. KGEMs were trained using PyKEEN’s hyperparameter optimization pipeline over 30 trials using as initial parameters the best configurations from Bonner *et al*. [14] for the models benchmarked in this study and Ali *et al*. [11] for the rest. The evaluation in the hyperparameter optimization was conducted using Hits@10 for all models on a link prediction task of drug–disease triples (see *Train, validation, and test datasets*). Details about the configurations are available at the GitHub repository (https://github.com/enveda/kgem-ensembles-in-drug-discovery).

### Ensemble methodologies

As previously mentioned, KGEMs can foster drug discovery by nominating a set of drug–disease pairs that have a higher chance of succeeding in clinical trials than one would expect by chance. Since only a limited number of pairs can be experimentally screened, KGEMs which achieve a high accuracy for the top predicted drug–disease triples are preferred over models that might achieve a better overall precision but exhibit a lower accuracy among the top predictions. Below, we propose and analyze different methodologies to combine the prediction scores from an ensemble of models to maximize the performance among the top predictions.

### Normalizing scores across models

**Figure 1:**
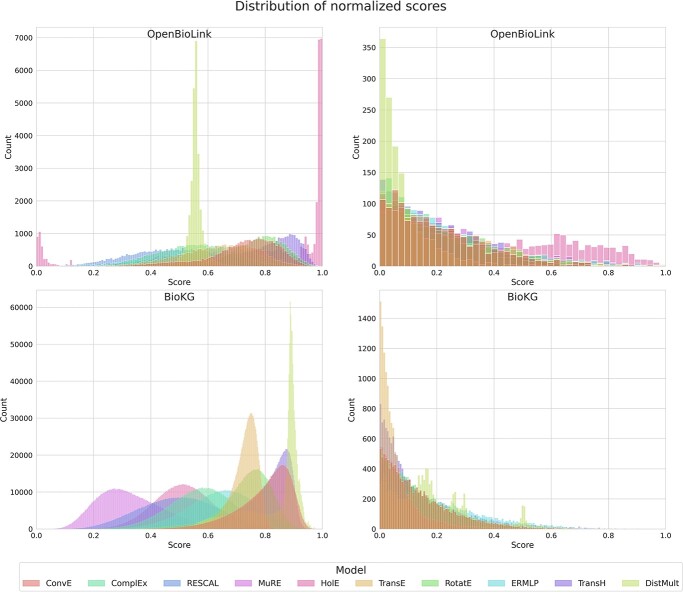
(Left) Score distributions for all drug–disease predicted triples after min-max normalization for each model on the two KGs. Right) Score distributions after normalizing the 99th percentile drug–disease triples (top predictions) for each model on the two KGs. For simplicity, scores have been normalized between 0 and 1. The score distributions are generated with the best trained model. By normalizing the top predicted triples (in the example the top 1%) as opposed to all predicted triples, we can generate a similar score distribution for each model while maintaining the distances between the scores. This in turn allows for better aggregation of the scores across models for a given triple. Note that the renormalized distribution of the top 1% triples is similar for each model despite large differences in distributions prior to the renormalization.

KGEMs return a score that represents the plausibility of a triple using embeddings for entities and relations. As they all have their own specific real-valued function, they produce different score distributions which typically lie in different intervals, as illustrated in Supplementary Figure 2. Therefore, to build an ensemble that aggregates plausibility scores from different KGEMs, scores must first be normalized on the same scale. Furthermore, the score distribution ultimately denotes the underlying model’s confidence on the predicted triples. For instance, a right-skewed distribution indicates that the model is exclusively confident about a minority of the predictions (right tail), as opposed to a normal distribution. Thus, given the varied distributions produced by different models, normalizing scores can provide comparable intervals under which ensemble models could operate. However, simply combining normalized scores implies weighing each model differently depending on its underlying distribution (e.g., left-skewed distributions would be weighted higher than right-skewed ones) (Figure 1; left). In addition, since the scores follow a Gaussian distribution (Supplementary Figure 3) and we are only interested in the extreme where the most confident predictions are (i.e., in the right tail of the distribution for a drug discovery task), normalizing based on the entire distribution masks the small differences observed among this extreme. These differences, albeit seemingly marginal compared to the entire distribution, are the most critical for the prioritization of one top predicted link over another. For instance, a model that yields a left-skewed distribution may have its top 1% predictions clustered within the same score interval, whereas the right-skewed distribution could have only a few top predictions.

For this reason, we propose selecting a predetermined number of top K predictions for each model and exclusively conducting the normalization on them. This not only solves the problem of giving different weights to models with different distributions, but also ensures that top predictions are weighted more than the rest by the ensemble approach and that differences among the top predictions are accentuated, both of which are desired properties in a drug discovery application.

Although other arbitrary thresholds could be applied, we focus on three conservative thresholds for the top K predictions (i.e., 5 1 and 0.1%). Here, it is important to note that the choice of the threshold has to be adjusted with the size of the KG as well as the top K to be prioritized (e.g., one may want to focus on the top 10 or top 500 predictions). Consequently, we set the baseline threshold at 5% (i.e., 95th percentile) for OpenBioLink and 1% for BioKG (i.e., 99th percentile) (results presented in *Ensemble models outperform individual KGEMs*). In the case of BioKG, this threshold was chosen because the number of drug–disease triples to be tested is significantly larger than OpenBioLink (Table 1), and corresponds to the top 13 502 predictions of each individual KGEM (among all 1 350 266 possible drug–disease triples between drugs and disease in the test set). On the other hand, given the smaller size of OpenBioLink, we increased the threshold to the top 5% predictions (i.e., 95th percentile), as a 1% threshold corresponds to <500 triples and thus, would not allow us to evaluate the ensemble model on K = 500. We evaluate the relative performance of applying all previously mentioned thresholds in Subsection *Ensemble models outperform individual KGEMs*.

Once the top predicted triples have been identified via thresholding, we can now normalize the prediction scores with the goal of preserving the relative distances between the scores of the top predictions. To achieve this, a variety of normalization methods can be applied. As a baseline, we employed min-max normalization, the most common normalization approach, which maintains the relative distance between the predicted scores. In addition, we tested sigmoid normalization, which yields an S-shaped distribution where scores are regularized on the low and high ends of the distribution. Finally, we evaluated min-max applied to the rank of the predicted link instead of the predicted score, which uniformly distributes the relative distance between triples (i.e., the difference between the normalized scores of the top 1 triple and the top 2 is the same as between the top 5000 and the top 5001). We evaluated the relative performance of applying each of these normalization methods in *Ensemble models outperform individual KGEMs*.

As an illustration, Figure 1 (right) shows the distributions of the normalized scores on both KGs after applying the 99th percentile threshold. The resulting distributions match different forms of an exponential distribution with different values of lambda.

### Generating the ensemble predictions

After normalizing the prediction scores for each model, scores must then be combined or aggregated to effectively leverage an ensemble of models. One of the most common techniques for this purpose is the majority voting algorithm, which combines the predictions of multiple models by considering the majority vote. In regression problems, this is achieved by averaging the values predicted by each model (also known as *soft voting*), whereas for classification problems, it involves summing the votes for classes predicted by each model and considering the class with the most votes (also known as *hard voting*).

Although the task of drug–disease prediction can be seen as a binary classification task in which a drug either does or does not *treat* a disease, it is framed as a regression problem in which KGEMs output a certain score or confidence for each triple and the Kth triple sets the threshold for which a drug is prioritized. Thus, a score can be treated as a threshold where a decision boundary is set and only predicted triples with a score above this threshold are considered. These triples can then be used to combine predictions from different models after normalization has been conducted (see subsection *Normalizing scores across models*). Consequently, ensemble predictions are calculated by aggregating (i.e., summing) the normalized scores for the top K triples. The common approach when aggregating using *soft-voting* is to apply the average to the scores predicted by all models. However, as we filter out part of the predicted triples, the precision resulting from applying the average (considering a score of 0 for filtered triples) is heavily penalized by models with poor performance, as they tend to prioritize a disjoint set of triples, which also tend to be false positives (i.e., triples not present in the KG) given their low precision. Furthermore, since disparate models will place different triples in the top K predictions, any triple not in the top K for a particular model is treated as if it was given a score of 0 by the model. This approach inherently gives more weight to frequently occurring triples which are predicted across more than one model. Although this will happen for both, true positives and false positives, we observed that different models tend to agree more on the true positives than on the false positives (Section *Investigating the agreement of the top predicted triples across different models*). In addition, we also tested the performance of the aggregation of averaged normalized scores. Note that the average is calculated only for normalized scores of predicted triples that are within the top 1 and 5%.

### Defining the ensemble models

As the ensemble model’s predictions depend on the quality of predictions by each of the individual models, if the majority of KGEMs incorrectly prioritize a drug–disease triple, the ensemble will most likely do so as well. Thus, to evaluate the robustness of our approach, we consider two different ensembles, each composed of different models.

- *Ensemble-top5*: composed of the top five best-performing models, as per their Precision@Top100 in the validation dataset.
- *Ensemble-all*: composed of all 10 benchmarked models.

Both ensemble models were tested using the thresholds and normalization approaches described in 3.5.1. Furthermore, we added two additional configurations using PoE [20] and an ensemble that prioritizes triples based on ranked positions instead of the predicted scores, referred to as *position norm.*

### Model evaluation

Since our goal is to assess the top-ranked predictions, we evaluated all models using Precision@K as a metric, which corresponds to the proportion of true positives within the top K drug–disease triples predicted by the model. To evaluate the models within a broad range, we selected typical values of K ranging from 1 to 500. We discarded exploring larger values of K as the number of positive triples in the OpenBioLink test set was 515 (Table 1) and we are conducting a drug discovery task where only the top-ranked predictions would be experimentally validated. We would like to note the difference between Precision@K, the chosen metric for model evaluation and Hits@K in the context of link-prediction. Although Precision@K indicates the fraction of triples present in the test set after ranking all drug–disease triples, Hits@K represents the average proportion at which a triple in the test set appears within the top K with respect to other corrupt triples.

## Results

This section begins by presenting a benchmark of 10 KGEMs on two biomedical KGs (subsection *Benchmarking the performance of models*) which revealed large differences in performance across models as well as identified the best performing KGEMs. Then, in subsection *Investigating the agreement of the top predicted triples across different models*, we investigated the overlap of the top predicted triples across different models, demonstrating that KGEMs tend to show a higher degree of agreement on triples that have been correctly prioritized. Prompted by these findings, in subsection* Ensemble models outperform individual KGEMs* , we showed how combining the predictions from several models through ensemble learning can outperform the best-performing KGEMs. Finally, in subsection *Investigating prioritized predictions by the ensemble model*, we explored a subset of the top predictions prioritized by the ensemble models that were not prioritized by any KGEM.

### Benchmarking the performance of models

**Figure 2:**
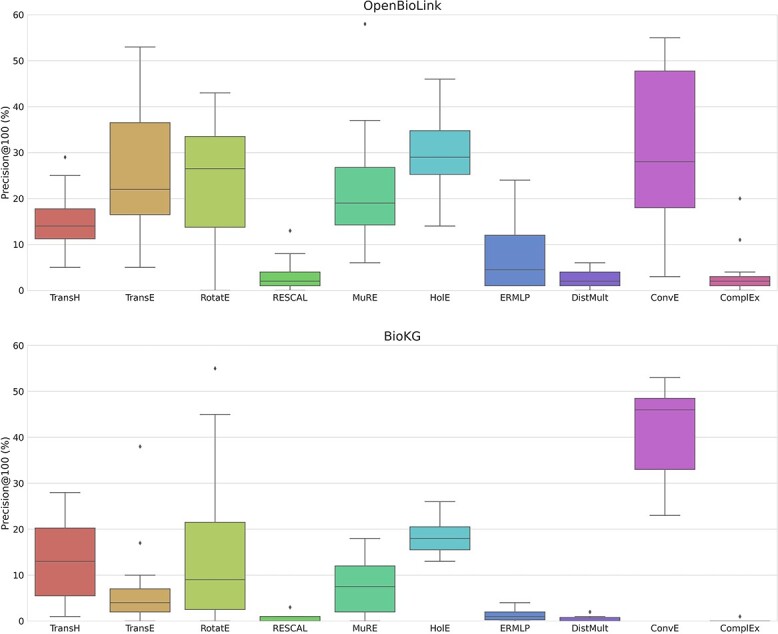
Distribution of the Precision@100 achieved for each model trained with different hyperparameters in the OpenBioLink and BioKG KGs. Note that this evaluation is conducted on the test set of both KGs. Supplementary Table 1 shows the performance of the best hyperparameters found for each model on both the validation and the test set.

We began by investigating the robustness of different training setups on the 10 benchmarked models, observing similar patterns on both KGs (Figure 2). ConvE, HolE and RotatE achieved the best performance, followed by MuRE, TransE and TransH. The remaining models (i.e., ERMLP, DistMult, RESCAL and ComplEx) achieved low performance on both KGs.

Notably, our results are consistent with recent benchmarks that included five of these models [13, 14]. For instance, similar to our results, both benchmarks reported a high performance for RotatE closely followed by TransE, and a low performance for ComplEx. When comparing the performance on the two KGs, we observed that the 10 benchmarked models exhibited significantly worse performance on BioKG compared with OpenBioLink with the exceptions of TransH and ConvE. Although in the case of TransH a similar median performance was achieved in both KGs, the median performance (Precision@100) of the trained ConvE models increased from 30 on OpenBioLink to 50 on BioKG (for reference, the second best performing model on BioKG was HolE with a median precision below 20). In addition, we investigated the performance of the best hyperparameters found (i.e., best model across all runs) on the validation and test dataset (Supplementary Table 2). Here, all models performed better on the test set than on the validation set. In OpenBioLink, MuRE achieved the best performance across all models (see outlier on the boxplot of MuRE for OpenBioLink in Figure 2) with a Precision@100 of 41 and 58 on validation and test sets, respectively. In BioKG, ConvE and RotatE achieved the best performance. Using Precision@10, the majority of the models obtain a higher precision, although the relative precision among them follows a similar trend (Supplementary Figure 4).

Taken together, three major conclusions can be drawn from our benchmark when using KGEMs for link prediction on biomedical KGs. The first one is the importance of model selection given that this and previous benchmarks [13, 14] have revealed significant differences in performances across models. The second is the high variability observed in the performance across runs, which highlights the importance of finding the best hyperparameters for each model-KG pair. Finally, not only is RotatE a top performer as identified in earlier benchmarks, HolE and in particular, ConvE, all exhibit superior performance for a link prediction task on biomedical KGs.

### Investigating the agreement of the top predicted triples across different models

**Figure 3:**
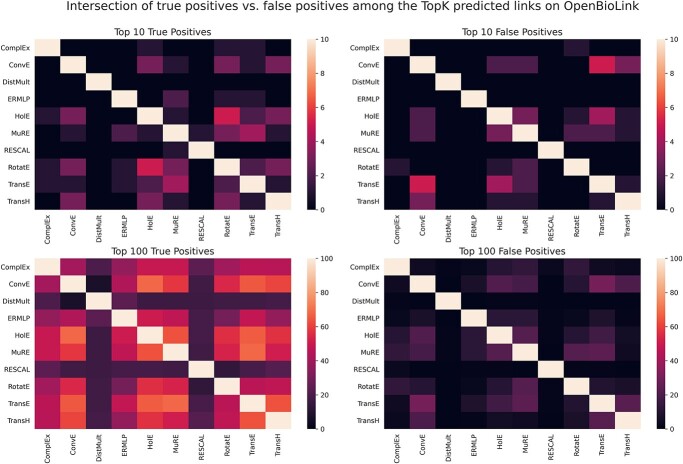
Pairwise intersection of the top 10 and top 100 drug–disease triples predicted by each model on OpenBioLink. The heatmaps are divided into the intersection of true positives (drug–disease triples in the test dataset) (left) and the intersection of false positives (right).

Given that each KGEM employs a different methodology to represent a KG, not surprisingly, differences are observable among top predicted triples, which are typically the most interesting ones to explore in a drug–disease prediction task. Prompted by this, we subsequently investigated the overlap of the top K-predicted drug–disease triples (excluding triples already seen by the model in training and validation) for the best performing hyperparameters of each model. Interestingly, we observed that the overlap of true positives is significantly larger than the overlap of false positives for the same value of K. As an illustration, Figure 3 depicts the intersection of the top 10 and top 100 predicted triples between drugs and diseases by the 10 models benchmarked on OpenBioLink. While the left part of the figure shows an overlap of approximately four times higher of % true positive triples for the top 100 compared with the overlap of false positives (right), which is almost nonexistent. The same trend is maintained across different Ks and BioKG (Supplementary Figure 5). These results suggest that KGEMs tend to agree more on correctly prioritized triples. Together, these experiments illustrate that although overall the models demonstrate minimal overlap between the top predicted triples, overlapping triples are more likely to be true predictions than false positives.

### Ensemble models outperform individual KGEMs

**Figure 4:**
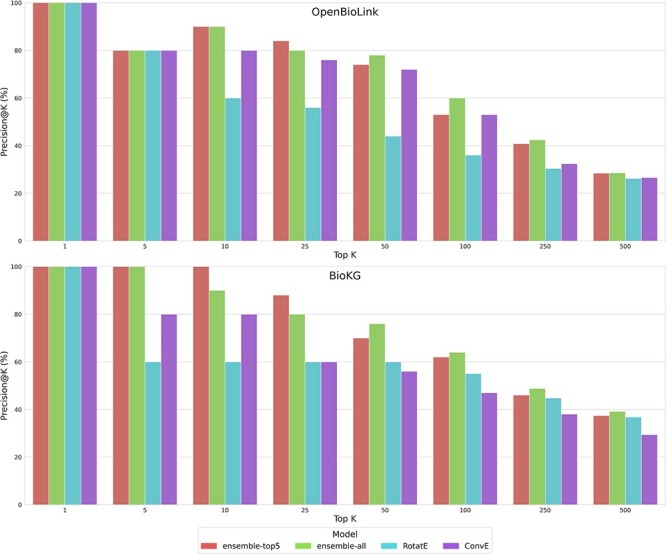
Precision at Top K in the test set using different values of K in the OpenBioLink and BioKG. For predefined values of K, the Precision@K for top predicted drug–disease triples are displayed for two ensembles (i.e., ensemble-all and ensemble-top5) and two independent KGEMs (i.e., RotatE and ConvE) using the 99th (BioKG) and 95th (OpenBioLink) percentile normalization approach. Although the latter two KGEMs represent the two best performing benchmarked models, the ensemble models outperform each of these individual models.

In this subsection, we compare the performance of the different ensemble models against individual KGEMs. Figure 4 illustrates the precision on the top K-predicted triples for the two baseline ensemble models (i.e., *ensemble-all* and *ensemble-top5* using min-max normalization of the top 1% triples for BioKG and the top 5% triples for OpenBioLink) compared against the best performing KGEMs in each KG.

We found that the baseline ensemble models outperformed each of the individual ones at all investigated K, highlighting the benefit of applying ensemble learning to KGEMs. In both KGs, although most of the top predicted triples were true positives for all models at K = 5, differences in performance became more pronounced as K increased up to the top 100 predicted triples (e.g., RotatE consistently exhibits a lower Precision@k from top 10 to top 100). Conversely, differences in performance between the ensembles and the best individual models diminished when evaluating a larger K (i.e., 250 and 500). This can be partially attributed to the fact that the precision of the models is inversely proportional to K (i.e., top predictions evaluated), which is expected given that the number of positive triples evaluated (i.e., drug–disease triples) is a small proportion in comparison to all negative drug–disease triples that could potentially be predicted by the models.

When comparing the two baseline ensemble models for each KG, we observed that the ensemble of the top 5 performing KGEMs (i.e., *ensemble-top5*) systematically performed better than the ensemble built out of all models (i.e., *ensemble-all*) up to K = 25. From K = 50 onwards, we observed a gain in performance for *ensemble-all*, despite having poorer performing models.

**Figure 5:**
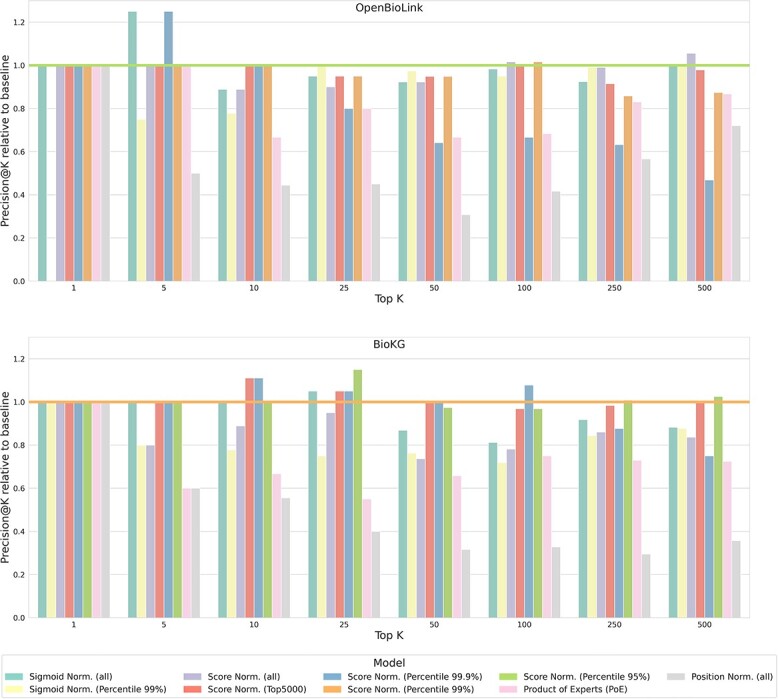
Precision at Top K using different values of K (i.e., top predicted drug–disease triples) comparing the different normalization approaches on the ensemble-all. The horizontal bar represents the performance of the baseline ensemble-all presented in Figure 4.

Figure 5 illustrates the effect of the selection of distinct thresholds and normalization approaches with respect to the baseline *ensemble-all*. PoE and the position-based ensembles achieved the lowest performance across all configurations. This is not surprising given that PoE is designed for and evaluated on a problem where most types of triples are of interest. Conversely, we are only interested in a small subset of triples (i.e., drug–disease triples), whereas KGEM models are biased towards predicting the most abundant type of triples, which correspond to protein–protein triples. With regard to the position-based ensemble, this model discards the underlying information held by the scores (i.e., the difference between two scores quantifies the relative confidence of the model for a given prediction), which can account for its relatively poor performance. In addition, another important aspect is setting an appropriate threshold as, for instance, the 99.9th percentile achieves the highest precision up to K = 25 in BioKG and K = 10 in OpenBioLink. However, because of this low threshold, the performance of this configuration quickly decays as K increases since the ensemble is focusing on prioritizing the very top triples. This suggests that this can be an effective threshold, if the goal is to focus on the top 10 or 50 triples. Furthermore, the sigmoid normalization, although for some specific values of K yields similar results as the baseline min-max normalization, generally achieved a considerably lower performance.

**Figure 6:**
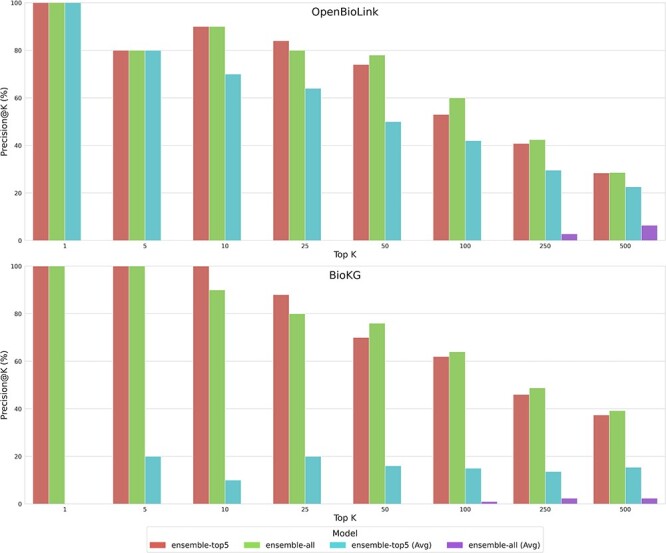
Precision at Top K using different values of K (i.e., top predicted drug–disease triples) comparing ensemble-all and ensemble-top5 using two score aggregation approaches: sum aggregation (baseline) and average (Avg0).

Lastly, we evaluate two approaches to combine the predictions of the different KGEMs into the ensembles. As shown in Figure 6, we found that the sum aggregation clearly outperforms the precision of the approaches using average aggregation since average aggregation is heavily penalized by KGEMs not prioritizing a given triple. Apart from the difference in precision observed between both approaches, it is important to highlight how the results of sum aggregation are not only robust, but the more models added to the ensemble seem to further improve precision, even if the added models perform poorly. This may be especially desirable because the performance of different KGEMs in previously unseen datasets will always be unknown, as we cannot possibly know beforehand which models will be the best or worst-performing models.

### Investigating prioritized predictions by the ensemble model

In this subsection, we sought to explore the top drug–disease triples predicted by the baseline *ensemble-all* model to better understand how reliable such predictions are compared with the individual models. Among the top 10 predictions of OpenBioLink, we found correctly predicted triples such as cyclophosphamide, fludarabine phosphate and busulfan, which were respectively predicted to treat neuroblastoma, chronic lymphocytic leukemia and myelodysplastic syndrome. Interestingly, despite the average predicted ranking for these three triplets being around 3000, they all end up in the top 10 (Table 2). For instance, in the case of the busulfan-myelodysplastic syndrome triple, the best ranking in an individual model is position 8 for HolE, but it is likely to be prioritized as it appears in the top 100 predicted triples for six out of the remaining nine models. A similar case occurs in BioKG, where, within the top five, atropine, carbamazepine and nifedipine are predicted to treat bradycardia, tremor and hypertension, respectively (Table 2). Finally, we also explored whether node degree correlates with the predicted score, as reported in Bonner *et al*. [12]. We observed a low correlation between all drug–disease pairs and their node degree for *ensemble-all*: 0.09 (BioKG) and 0.27 (OpenBioLink) (Supplementary Figure 6).

**Table 2:** Examples of the top 10 predicted triples by the baseline *ensemble-all* on both KGs

**KG**	**Drug**	**Disease**	**Ensemble ranking**	**Highest ranking in an individual model**
**BioKG**	Cyclophosphamide (pubchem.compound:2907)	Neuroblastoma (DOID:769)	4	5 (TransE and MuRE)
	Fludarabine phosphate (pubchem.compound:30751)	chronic lymphocytic leukemia (DOID:1040)	6	3 (ConvE)
	Busulfan (pubchem.compound:2478)	myelodysplastic syndrome (DOID:0050908)	8	8 (HolE)
**OpenBioLink**	Carbamazepine (drugbank:DB00564)	Tremor (mesh:D014202)	1	5 (ConvE)
	Atropine (drugbank:DB00572)	Bradycardia (mesh:D001919)	2	31 (HolE)
	Nifedipine (drugbank:DB01115)	Hypertension (mesh:D006973)	5	32 (RotatE)

## Discussion

As the variety of KGEMs have increased, so have the number of possible biomedical applications for KGEMs, driving the need for comparative studies that investigate the performance of disparate models across various downstream tasks. Here, we focused on a particular application in drug discovery (i.e., prediction of novel drug–disease triples) and benchmarked 10 distinct KGEMs on two biomedical KGs. Our results revealed large variability among the top predictions across both axes (i.e., KGEMs and KGs), highlighting the importance of the KGEM choice for this task. Furthermore, we also observed a low overlap of true positives among the top predictions, which prompted us to explore the use of ensemble models. Subsequently, we developed distinct ensemble methodologies that incorporate predictions from several KGEMs and included these ensemble models in our benchmark. These generated ensemble models systematically outperformed each of the individual KGEMs within the ensemble on two KGs, even when some KGEMs exhibited a significantly lower performance than the rest. In conclusion, our findings demonstrate the benefits of ensemble approaches that combine predictions from several KGEMs.

One of the major challenges in predicting drugs for a given disease is that the majority of drug–disease combinations have yet to be explored, resulting in a scarcity of data for validation and imperfect definitions for negative labels. This can lead to significant differences in the precision of the same model when evaluated on different biomedical KGs [14]. We attempted to mitigate this factor by including two KGs in our benchmark which share equivalent triples but vary in size. Furthermore, we followed a similar strategy as Ratajczak and colleagues [10] by training the KGEMs using validation and test splits that exclusively contained drug–disease triples. Outstanding questions remain regarding the performance of other non-benchmarked models and the inclusion of novel KGs. With respect to the former, we envision a future benchmark of KGEMs for drug–disease link prediction on other biomedical KGs, such as Hetionet [35] and CKG [36], that could potentially corroborate our findings. These KGs significantly differ from the two selected ones as they contain several additional nodes (e.g., pathways, anatomical regions) and edge types, as well as several millions of triples. This constituted the primary reason why these KGs were not included in our benchmark as we intended to conduct a comparison across KGs that contained the same node and relations types. Similarly, although here we have focused on 10 of the most widely used KGEMs, other KGEMs beyond the ones employed in our study could also be used. It is worth mentioning that a known limitation of KGEMs is that they generally attribute higher scores to overrepresented entities (nodes with a higher degree) [12]. Thus, future work could focus on developing more advanced ensemble models that take this bias into account. In addition, although our benchmark was consistent with previous work [13, 14], we would like to highlight two important differences between our and their evaluation. First, they employed a different evaluation metric than ours (i.e., Hits@10). Second, although Bonner *et al.* [14] also employed BioKG, their version of this KG included other node and edge types as they conducted a gene–disease prioritization task, as opposed to our drug–disease prediction task which made us reduce the KG to drugs, proteins and diseases. Finally, although our work has demonstrated that ensemble models can potentially yield better predictions, we would like to acknowledge that they require significantly more resources as several models have to be trained.

Lastly, it is important to note the differences between drug discovery applications (e.g., predicting drug–disease triples), in comparison to generic link prediction tasks on non-biomedical KGs. As emphasized in this introduction, biomedical applications generally require models with high accuracy for their top predicted triples, as opposed to a model with better overall performance across all evaluated triples. This is further exacerbated in drug discovery as the number of tested drugs tends to be limited. Thus, this benchmark focuses on the top predicted drug–disease triples, and although our results indicate that ensemble models outperform individual ones, results cannot necessarily be extrapolated beyond the characteristic of the study.

**Key Points**

- In recent years, the increasing number of KGEMs has led to several benchmarks to evaluate their performance in several biomedical KGs
- We evaluate 10 state-of-the-art KGEMs on a drug discovery task (i.e., predicting triples between drugs and diseases) to illustrate the large difference in performance across models
- To mitigate these differences and generate more robust predictions, we propose to adopt concepts of ensemble learning
- We demonstrate how combining the predictions of several models, despite their differences in performance, systematically yields better predictions

## Supplementary Material

Supplementary_File_bbac481

## Author Biographies

**Daniel Rivas-Barragan** is a researcher with expertise in big data, machine learning and optimization problems.

**Daniel Domingo-Fernández** is a senior data science manager at Enveda specialized at the intersection of biomedical data and knowledge for applications in drug discovery.

**Yojana Gadiya** is a researcher specialized in data science and data mining, particularly using Knowledge Graphs.

**David Healey** is VP of Data Science at Enveda and has an expertise in machine learning, metabolomics and computational biology.
