# Supplementary material for: Ensembles of knowledge graph embedding models improve predictions for drug discovery
Source: Brief Bioinform. 2022 Nov 16;23(6):bbac481. doi: 10.1093/bib/bbac481 (PMC9677479; doi:10.1093/bib/bbac481)
Supplement: Supplementary_File_bbac481 [file supplementary_file_bbac481.pdf]

Supplementary Figures

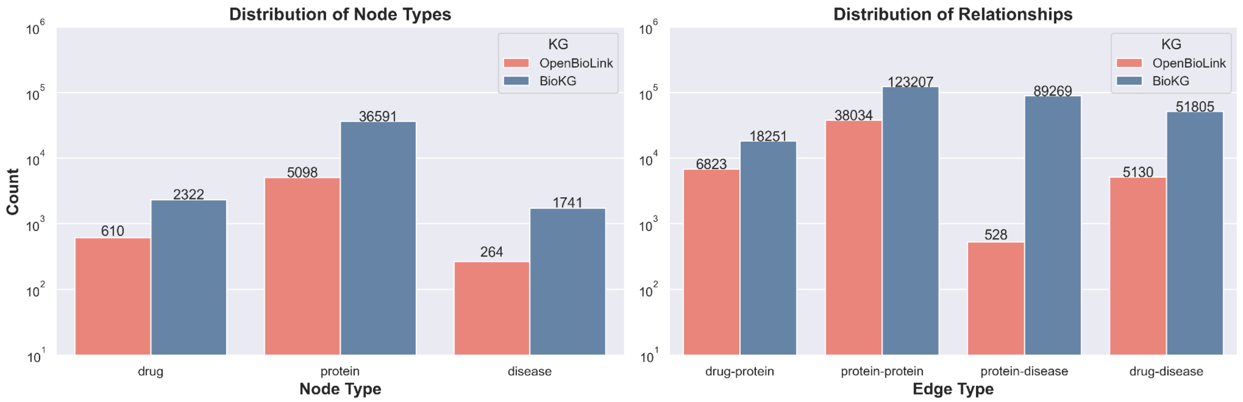

Supplementary Figure 1. Distribution of node and edge types in BioKG and OpenBioLink. Note that for the drug-protein, protein-protein, and protein-disease edge type, there are three different relations: increase, decrease, and association, depending on the KG. However, drugs and diseases are exclusively connected by one relation (treats).

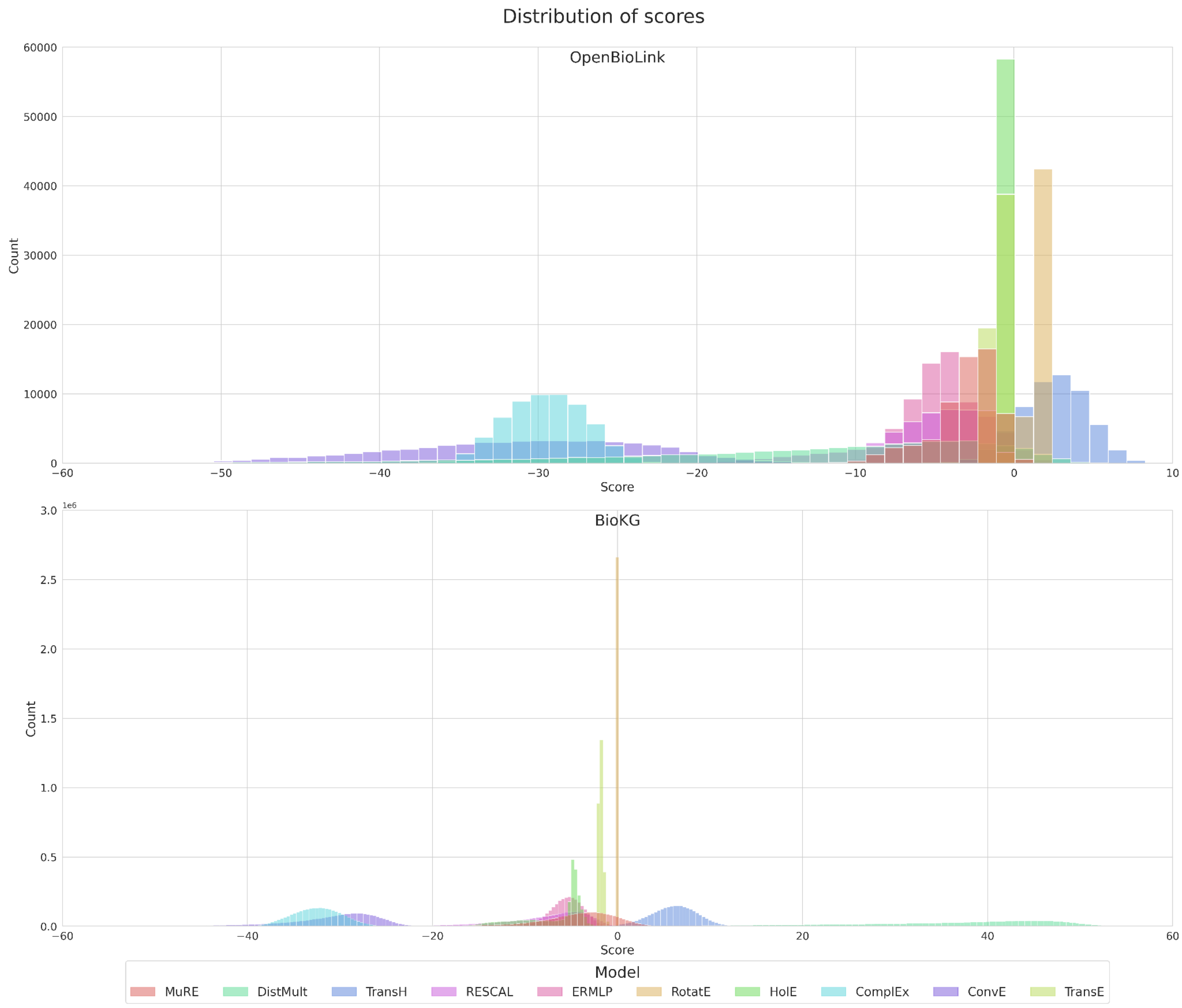

Supplementary Figure 2. Original score distributions for all possible drug-treats-disease triples (excluding those present in the training or validation sets) for each model over the two benchmarked KGs.

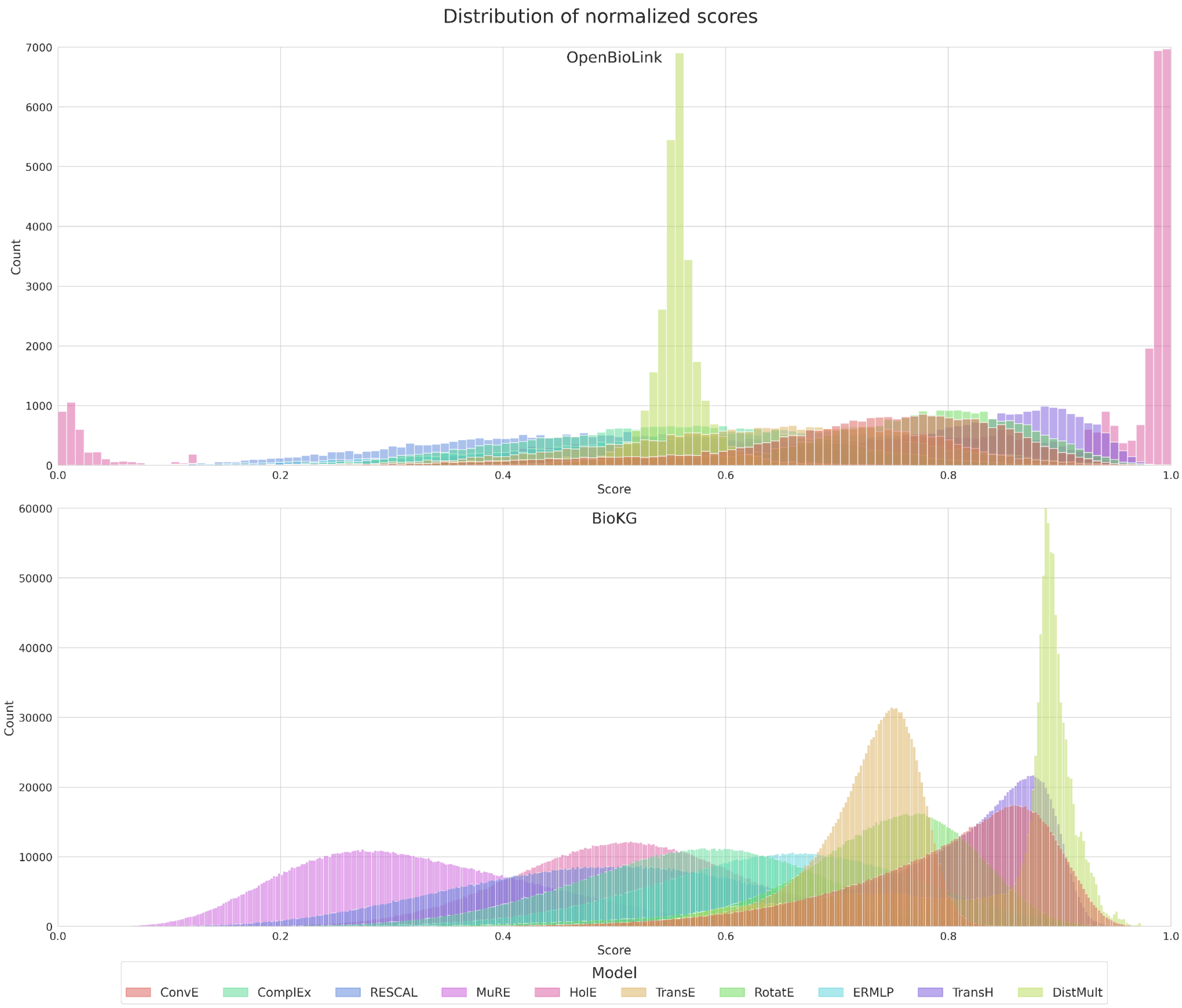

Supplementary Figure 3. Normalized score distributions between 0 and 1 for all possible drug-treats-disease triples (excluding those present in the training or validation sets) for each model over the two benchmarked KGs.

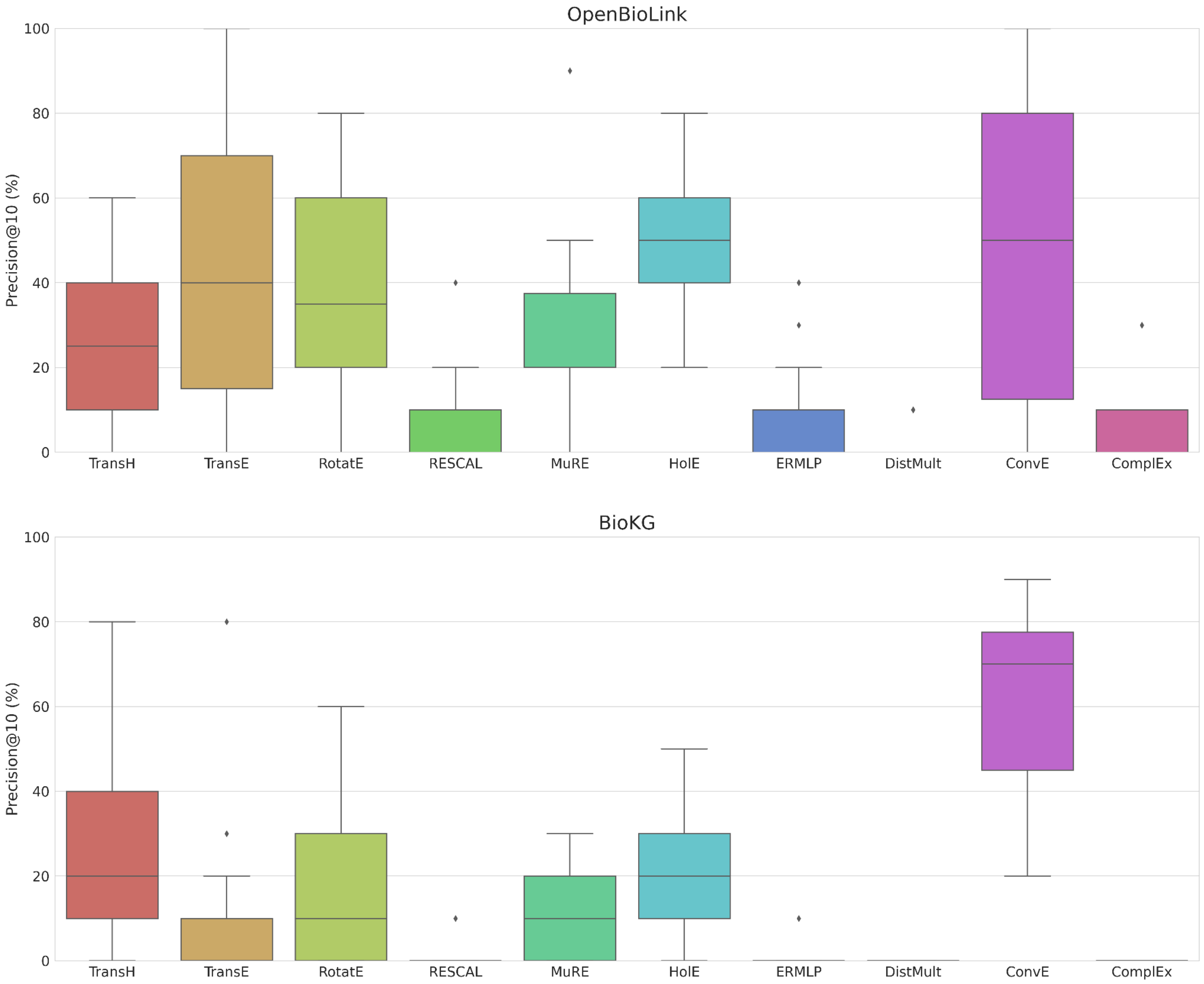

Supplementary Figure 4. Distribution of the Precision@10 achieved for each model trained with different hyperparameters in both KGs.

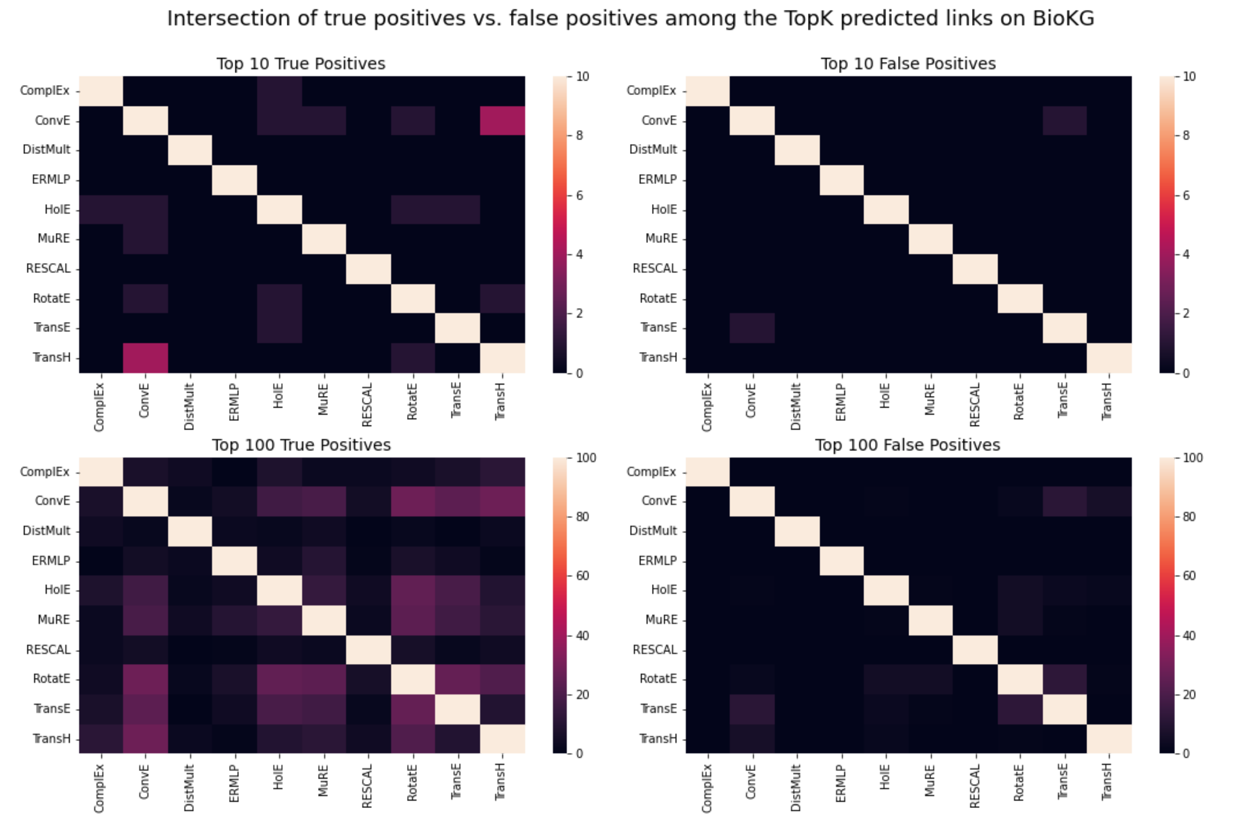

Supplementary Figure 5. Pairwise intersection of the top 10 and top 100 drug-disease triples predicted by each model on BioKG. The heatmaps are divided into the intersection of true positives (drug-disease triples in the test dataset) (**left**) and the intersection of false positives (**right**).

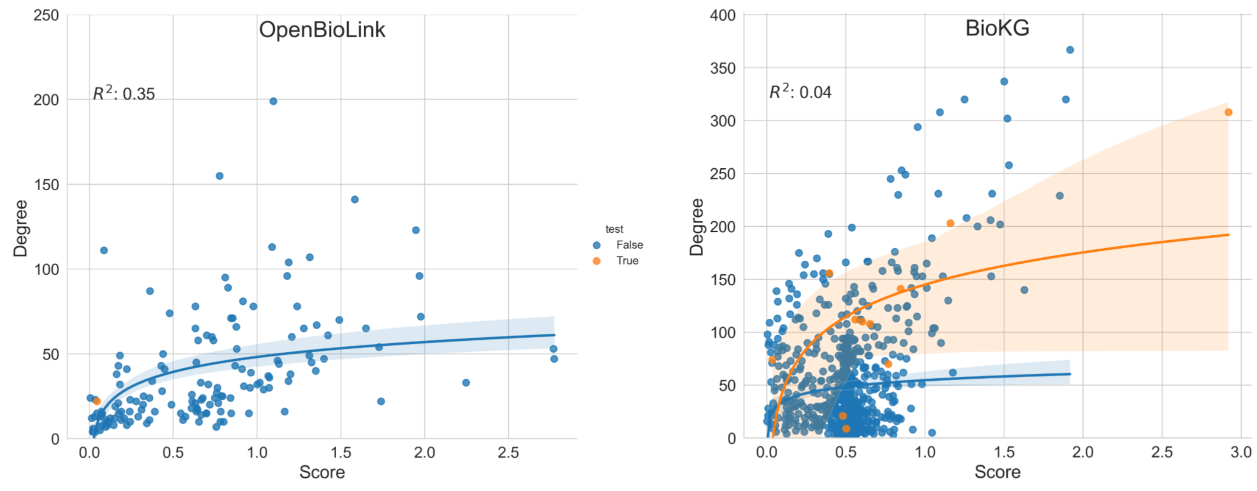

Supplementary Figure 6. Relation between the node degree and drug-disease triple predictions for two showcase disorders from each KG. The plot shows the association between the degree of all the drug nodes and their predicted score by the *ensemble-all* model for liver cirrhosis in Openbiolink (**left**) and alcohol use disorder in BioKG (**right**). Although these are two disease examples, the average correlation for all diseases was similar: 0.09 (BioKG) and 0.27 (OpenBioLink).

Supplementary Tables

Model	Type	Loss function	Reference
ComplEx	Tensor factorization	NSSALoss	(Trouillon *et al.*, 2016)
RESCAL	Tensor factorization	BCEAfterSigmoidLoss	(Nickel *et al.*, 2011)
DistMult	Tensor factorization	MarginRankingLoss	(Yang *et al.*, 2014)
ConvE	Neural Network	BCEAfterSigmoidLoss	(Dettmers *et al.*, 2018)
ERMLP	Neural Network	MarginRankingLoss	(Dong *et al.*, 2014)
MuRE	Geometry model	BCEWithLogitsLoss	(Balazevic *et al.*, 2019)
RotatE	Geometry model	NSSALoss	(Sun *et al.*, 2019)
HolE	Translation model	MarginRankingLoss	(Nickel *et al.*, 2016)
TransE	Translation model	BCEWithLogitsLoss	(Bordes *et al.*, 2013)
TransH	Translation model	MarginRankingLoss	(Wang *et al.*, 2014)

Supplementary Table 1. Knowledge Graph Embedding Models benchmarked in our work. The second column presents the type of KGEM. The third column indicates the loss function used. The four column is the reference to the original paper describing the model. Types are taken from Linjuan *et al*. (2022). "Knowledge Graph Embedding Based on Semantic Hierarchy".

Model	KGs			
	BioKG		OpenBioLink	
	Validation	Test	Validation	Test
**RESCAL**	3%	3%	0%	0%
**TransE**	26%	38%	38%	53%
**DistMult**	1%	1%	4%	5%
**ERMLP**	1%	2%	14%	24%
**TransH**	20%	23%	23%	25%
**ComplEx**	0%	0%	16%	20%
**HolE**	18%	26%	31%	46%
**ConvE**	**36%**	47%	40%	53%
**RotatE**	35%	**55%**	27%	37%
**MuRE**	12%	13%	**41%**	**58%**

Supplementary Table 2. Validation and test Precision@100 of the different KGEMs using the best trained model. We would like to note that the evaluation is conducted on the top 100 predicted drug-disease triples. This table complements the results of Figure 3 by showing the performance on the validation set as well. From the results shown in this table, it is important to notice two important aspects. First, if only one KGEM were to be picked, the choice varies depending on whether the model is evaluated on the validation dataset or the test dataset. For the two KGs on which the KGEMs were evaluated, the best-performing model differs when the dataset changes. In a real-life scenario, the test dataset would be equivalent to the dataset of unseen triples we are interested in predicting. Therefore, by choosing a single model, there is never a guarantee that it would make up for the optimal choice. Second, there is a non-negligible disparity among the precision the different KGEMs may achieve. Consequently, we tested what happens when we do not consider the predictions of those models clearly underperforming.

Drug identifier (DrugBank)	Disease identifier (MeSH)	Positive triple	Ensemble all	conve	rescal	complex	rotate	ermlp	hole	transe	transh	mure	distmult
**DB00945**	**D014839**	True	0	149	169493	87521	49	18177	5586	920	279	356	25
**DB00564**	**D014202**	True	1	5	61798	29813	80	37540	545	59	737	76	819967
**DB00572**	**D001919**	True	2	33	1395	87246	86	15202	31	168	9820	209	510290
**DB00945**	**D005334**	True	3	129	255617	13367	245	4777	9263	108	417	1	144361
**DB00252**	**D012640**	True	4	2	188274	22352	10	116726	1033	1084	9	26403	697226
**DB01115**	**D006973**	True	5	138	2212	82550	32	20947	197	540	1261	1398	1281526
**DB00635**	**D005334**	True	6	57	829866	65853	111	23876	840	33	468	815	1322304
**DB00515**	**D007674**	True	7	7	56559	236940	33	22228	8292	457	115	423	108149
**DB00363**	**D001919**	True	8	52	748400	15977	55	33165	2404	5	1522	269	361055
**DB00635**	**D017114**	False	9	37	705597	123020	246	84745	452	22	2259	414	1200472
**DB00860**	**D004342**	False	10	529	1069041	20539	455	10457	138	89	642	407	1027901
**DB00997**	**D009336**	True	11	103	30460	49500	215	49490	5735	182	23	544	890473
**DB00563**	**D014839**	True	12	6	187046	68597	340	34661	9	24017	44	2839	688101
**DB01234**	**D006973**	True	13	4	800549	20954	29	129336	5471	4300	1	3251	1197044
**DB00281**	**D006323**	True	14	1333	319199	11956	131	3171	22	9	13423	288	1224276
**DB00328**	**D005334**	True	15	131	556089	46069	7	55716	32554	29	521	591	57031
**DB00313**	**D056486**	True	16	13	9820	3980	14	61604	88	10717	123	30492	790958
**DB00328**	**D064420**	False	17	61	377600	49831	980	64229	46	55	257	31670	30767
**DB00813**	**D001919**	True	18	67	102726	19879	75	9995	6	520	12532	1023	105671
**DB00907**	**D014839**	True	19	8	168876	1944	778	10442	11901	99	20	4351	1202895
**DB00091**	**D004487**	False	20	856	992528	48066	134	53646	249	70	2185	164	1323753
**DB00945**	**D007674**	True	21	45	29600	50012	79	6394	1859	413	190	6482	28998
**DB04216**	**D007249**	True	22	867	88230	17387	9	18512	98	132	1344	6468	1276568
**DB02709**	**D008106**	True	23	3290	373	143236	121	95543	943	12722	27	354	76996
**DB00363**	**D006261**	False	24	225	796243	9708	6	23368	995	114	1880	3192	958385

Supplementary Table 3. Top 25 predicted drug-disease triples by the baseline *ensemble-all* for BioKG. The first two columns correspond to the drug and disease as well as their identifiers. The third column indicates if the triple was accurately predicted (i.e., it is present in the test set). The remaining columns indicate the position of the score by the ensemble-all and the individual KGEMs.

Drug identifier (PubChem ID)	Disease identifier (DOID)	Positive triple	Ensemble all	conve	rescal	complex	rotate	ermlp	hole	transe	transh	mure	distmult
**30751**	**707**	True	0	7	12123	329	4	49	1	10	49	11	12808
**2907**	**0050908**	True	1	12	11816	66	3	1029	0	26	0	12	12746
**460612**	**0060058**	False	2	6	7436	62	37	994	4	27	9	19	12924
**2907**	**4235**	True	3	32	8690	199	34	537	2	14	4	95	11751
**5743**	**162**	True	4	1	19585	957	0	925	6	29	71	39	10445
**2478**	**0060060**	True	5	16	21534	210	15	347	62	23	28	44	14046
**2907**	**769**	True	6	18	18914	2046	21	502	36	5	19	5	13604
**30751**	**3721**	True	7	52	15695	1015	22	183	5	1	115	16	8622
**2478**	**8432**	True	8	137	9738	441	5	226	37	0	343	6	9716
**36462**	**2394**	True	9	49	20891	0	380	56	46	37	443	27	11385
**5865**	**2394**	False	10	4	23497	256	52	197	63	8	120	73	13192
**30751**	**1040**	True	11	3	15852	2178	62	117	40	16	42	18	12725
**2478**	**0050908**	True	12	37	13882	159	14	1223	8	53	14	53	13255
**5426**	**9119**	False	13	24	27815	332	64	187	13	35	113	35	9473
**3690**	**0060058**	True	14	41	26867	80	2	242	70	68	323	36	13129
**5426**	**8552**	False	15	120	16706	325	23	45	14	43	409	30	10429
**5865**	**0050908**	True	16	34	13964	923	129	642	24	7	29	26	12153
**6741**	**10923**	True	17	315	19756	195	385	150	41	78	57	75	744
**2478**	**1588**	True	18	231	8042	142	19	1249	153	18	61	1	8598
**30751**	**1588**	True	19	276	13799	1332	31	154	47	15	82	2	6225
**5743**	**707**	True	20	47	23273	14	65	3881	191	11	199	38	11098
**126941**	**0060060**	True	21	59	22745	1632	150	371	20	21	97	13	11275
**126941**	**9080**	True	22	45	17943	1747	171	21	232	9	317	8	9614
**5284373**	**8552**	True	23	36	15877	606	656	80	50	19	119	54	12805
**5978**	**2394**	False	24	55	6482	1895	32	64	10	131	386	47	11063

Supplementary Table 4. Top 25 predicted drug-disease triples by the baseline *ensemble-all* for OpenBioLink. The first two columns correspond to the drug and disease as well as their identifiers. The third column indicates if the triple was accurately predicted (i.e., it is present in the test set). The remaining columns indicate the position of the score by the ensemble-all and the individual KGEMs.
